# Immunization status of children with cerebral palsy: A cross-sectional hospital-based study in Vietnam

**DOI:** 10.1371/journal.pone.0323081

**Published:** 2025-05-07

**Authors:** Thi Hong Hanh Khuc, Tasneem Karim, Minh Chau Cao, Thi Van Anh Nguyen, Thi Huong Giang Nguyen, Quang Dung Trinh, Rachael Dossetor, Van Bang Nguyen, Nadia Badawi, Lal Rawal, Gulam Khandaker, Elizabeth Jane Elliott

**Affiliations:** 1 School of Health, Medical and Applied Sciences, Central Queensland University, Rockhampton, Queensland, Australia; 2 Faculty of Medical Technology, Phenikaa University, Hanoi, Vietnam; 3 Cerebral Palsy Alliance Research Institute, Specialty of Child & Adolescent Health, Faculty of Medicine and Health, The University of Sydney, Sydney, New South Wales, Australia; 4 Children’s Hospital Westmead Clinical School, Specialty of Child and Adolescent Health, Faculty of Medicine and Health, The University of Sydney, Sydney, New South Wales, Australia; 5 CSF Global, Dhaka, Bangladesh; 6 Asian Institute of Disability and Development (AIDD), University of South Asia, Dhaka, Bangladesh; 7 Medical Education and Skills Laboratory Department, Hanoi Medical University, Hanoi, Vietnam; 8 Rehabilitation Department, National Children’s Hospital, Hanoi, Vietnam; 9 Pediatric Department, Medical Faculty, Phenikaa University, Hanoi, Vietnam; 10 Grace Centre for Newborn Intensive Care, The Children’s Hospital at Westmead, Sydney, New South Wales, Australia; 11 Central Queensland Public Health Unit, Central Queensland Hospital and Health Service, Rockhampton, Queensland, Australia; 12 Kid’s Research, The Sydney Children’s Hospitals Network (Westmead), Sydney, New South Wales, Australia.; Sohag University Faculty of Medicine, EGYPT

## Abstract

**Aim:**

To describe the immunization status of children with CP in Vietnam and identify factors associated with vaccine non-uptake in this group.

**Methods:**

We conducted an active prospective case ascertainment of children with cerebral palsy (CP) attending the National Children’s Hospital in Hanoi between June to November 2017, following the model proposed by the Paediatric Active Enhanced Disease Surveillance system in Australia. All children were assessed by trained paediatricians at the hospital and their immunization history was recorded.

**Results:**

Data were collected from 765 children with CP (median age = 1.7 years, IQR = 2.7 years). Of these children, 82.7% were fully immunized for their age (compared to 96.4% of the general child population) according to the Vietnamese Expanded Programme on Immunization (EPI) schedule. A BCG vaccination scar was present in 94.0% of children with CP, and 95.9% of eligible children had received the measles-rubella vaccine as part of the national campaign (compared with 96.0% and 98.2% of the general population respectively). Incomplete vaccination according to the EPI was associated with younger age, living in an earth/sand house, homebirth, low-level maternal education, being diagnosed with CP before the age of three, having bilateral CP, having associated impairments (i.e., epilepsy, intellectual, visual, speech), being at level IV-V on the Gross Motor Function Classification System, and being undernutrition.

**Conclusion:**

This is the first study to document the immunization status of children with CP in Vietnam. A large proportion had not received the measles-rubella vaccine and 17.3% were not fully immunized. To increase vaccination coverage, interventions and strategies are required to ensure that all children with CP have equitable access to early diagnosis, immunization, health education programs, outreach programs, and frequent follow-up. Early diagnosis and focused intervention in early life could further improve vaccination coverage in children with CP.

## Introduction

Childhood immunization is recognised as one of the safest, most cost-effective and successful public health interventions to save lives and prevent disability[1]. Every child has the right to health and access to immunization[2,3] and the Expanded Programme on Immunization (EPI) was launched in 1974 to ensure that children worldwide are protected against vaccine-preventable diseases. Today, every country has a national immunization programme[1].

Vaccine-preventable diseases can cause disability, and congenital or perinatal infection may result in cerebral palsy (CP)[4–6]. Children with a disability, including cerebral palsy (CP), are more vulnerable to the adverse effects of vaccine-preventable diseases[7]. They are often at significantly higher risk of hospitalization and complications from preventable infections compared to their peers without disability[8]. Hence, timely vaccination according to the recommended schedule is important to prevent severe disease and premature deaths for children, particularly those with disabilities such as CP. In a population-based study of children with CP in Bangladesh, most deaths were due to potentially preventable causes[9]. A systematic review of studies conducted in low and middle-income countries (LMICs) underscores the profound impact of infections on childhood disability, most stemming from vaccine-preventable diseases[10]. Therefore, immunization in early childhood is a key component for prevention of disability and can prevent a proportion of CP caused by vaccine-preventable infections.

A recent systematic review found that children with disabilities face significant barriers to immunization[11], including poverty and limited healthcare access. It highlighted that 19.2% of children with CP were not fully immunized, compared to 6.4–8% of children without disabilities[12]. The review also noted that most studies lacked clear definitions of disability, with only two studies from LMICs, Ecuador[13] and Nigeria[14]. Children with disabilities in impoverished communities are more vulnerable to infections[13] due to health disparities. Childhood immunization as part of primary health care is also a surrogate of access to health care and health equity[15] in LMICs. In Vietnam, we established a cohort of over 700 children newly diagnosed with CP at the National Children’s Hospital in Hanoi and aimed to assess their immunization status and identify factors associated with non-vaccination.

The EPI was introduced in 1981 in Vietnam[16]. It has become one of the strongest national immunization program in the Southeast Asian region with high uptake of all vaccines included in the schedule [17]. Since 2012, 100% of communes and wards nationwide have been covered by the EPI[16]. During the most recent Comprehensive Multi-Year Plan cycle (2014–2015) the measles-rubella (MR) vaccine was implemented[17]. In 2019, 96.4% children were fully immunized against the six key diseases (diphtheria, tetanus, pertussis, polio, tuberculosis and hepatitis)[18]. The proportion of children in the general population who received the MR vaccine was 98.2% (2015)[19] and in 2019 over 90% had received the BCG vaccine[18,20].

To the best of our knowledge, no information is available on immunization rates among children with CP in Vietnam. Data from the Victorian Cerebral Palsy Register and the Australian Childhood Immunization Register (ACIR) showed that 80.8% of Australian children with CP were fully immunized[12]. Immunization coverage in children with CP and other disabilities varies internationally, for example for BCG (73.1% - 98.4%)[21–24] and measles- rubella (43.2% - 90%)[12,21–23,25]. Data from two LMICs show that in Ecuador 96.9% of children with disabilities were fully immunized[13] and in Nigeria, 68.3% children were fully immunized[26].

The literature identifies various factors which influence uptake of immunization in children in the general population, including parental factors such as the mother’s educational level[27–30] and gestational age at the time of delivery[29], father’s occupation[29], health status of the child at the time of immunization[31], receipt of antenatal care[27,29], geographic location[29], birth order[29], place of birth[28], socioeconomic status and difficulty in reaching a health facility[21,27]. Among children with CP, the child’s age[12], severity of motor impairment[12], poor household sanitation[21], diagnosis of CP after the age of 3 years[21], cost of travel to the nearest clinics, cultural beliefs amongst families and health care workers regarding the importance of immunization for children with disabilities[21], and vaccine hesitancy from parents and physicians[32] were identified as factors associated with immunization status. The aim of this study was to fill an evidence gap and identify whether similar factors affect vaccine uptake in children with CP in Vietnam, and whether uptake is influenced by socioeconomic disparities or the healthcare system context in Vietnam? Understanding barriers to immunization among Vietnamese children with CP will inform strategies to increase vaccination coverage.

## Methods

### Study site and population

This study is part of a hospital-based surveillance study of children with cerebral palsy in Vietnam conducted at the National Children’s Hospital (NCH) in Hanoi, Vietnam. Detailed study methodology is described in the study protocol[33]. The clinical characteristics of the study cohort and their nutritional status have also been previously published[34]. In brief, consecutive children with CP presenting to NCH were identified and underwent a series of assessments.

### Clinical assessment and data collection

Diagnosis of CP was confirmed by experienced paediatricians following history taking, clinical assessment, review of existing hospital records and investigations using our study protocol. The data collected was based on that from the Paediatric Active Enhanced Disease Surveillance (PAEDS) system operating in Australia[33]. The definition, inclusion and exclusion criteria used for CP are described in the protocol[33]. The following procedures were used to collect the data:

#### Immunization status.

Immunization records were reviewed by the paediatricians when available. When immunization records were not available, paediatricians relied on parent/caregiver report and the presence or absence of the BCG scar, which is often used as a proxy for the BCG vaccine. If both the immunization record and reliable parental recall were unavailable, the child’s vaccination status was recorded as unknown.

A child was considered fully immunized, if s/he was appropriately immunized for age according to the Vietnam EPI schedule: Birth (BCG and HepB); 2, 3, and 4 months (DPT-HepB-Hib and Oral Polio Vaccine); 9 months (Measles); 18 months (Measles-Rubella). The Japanese Encephalitis Vaccine is given in 2 doses at 1 year of age (2 weeks apart) with a 3^rd^ dose one year later. A child was considered incompletely immunized if s/he was not fully immunized according to age and in accordance with Vietnam EPI schedule. Children aged 1–14 years who received the MR vaccine during the 2015 national MR campaign[35] (the most recent campaign) and up till 2017) were recorded. Presence of a BCG vaccination scar (usually on the left deltoid) was recorded.

#### Description of cerebral palsy.

Detailed clinical assessment was conducted and the type and topography of CP was identified[34]. The Gross Motor Function Classification System (GMFCS) was used to classify and describe motor function[36].

#### Assessment of risk factors.

Data related to the risk factors for CP were collected through history taking, review of hospital records and clinical assessment. Maternal febrile illness was defined as self-reported fever during pregnancy with a temperature ≥37.8°C. CP timing was categorized as prenatal/perinatal, postneonatal, or unknown if no clear cause was identified. Perinatal asphyxia was defined as the newborn failing to cry, delayed breathing (>1 min), or needing assistance. Non-physiological jaundice was physician-confirmed.

Information included maternal febrile illness during pregnancy, gestational age at birth, mode of delivery, birth weight of children, perinatal asphyxia, neonatal jaundice, maternal infection during pregnancy, consanguinity, multiple births and was reported previously[34].

#### Assessment of associated impairments.

The presence and severity of associated impairments (i.e., epilepsy, intellectual problems and visual, hearing or speech impairments) were documented based on clinical history, detailed clinical assessment conducted by paediatricians, report by parents/primary caregivers according to the Washington Group conceptual framework and review of relevant medical reports.

Intellectual impairment was assessed using clinical history, caregiver reports, and medical records, with pediatricians conducting evaluations when needed. Based on DSM-5 criteria[20] and American Association on Intellectual Developmental Disabilities[37], severity was classified as mild to moderate for slower development and severe for major delays in intellectual and adaptive functioning. Visual impairment was assessed using history, clinical evaluation, and WHO guidelines, classifying it as normal, mild to moderate, or severe (blindness)[38]. Hearing impairment was evaluated through history, examination, and otoscopic assessment, following WHO criteria[39]: normal, mild to moderate, or severe (deafness), though audiology was not performed. Speech assessment relied on caregiver history and medical evaluation, categorizing impairment as normal, mild to moderate, or severe (non-verbal). We described details of assessments and reported nutritional status previously[40].

#### Nutritional assessment.

Anthropometric measurements (height and weight) were documented to assess the nutritional status of children using a World Health Organization (WHO) guideline[41]. The nutritional status of children with CP was assessed using weight-for-age (WAZ), height-for-age (HAZ), and weight-for-height (WHZ) z-scores, calculated with WHO Anthro and AnthroPlus software. WAZ was measured for children under 121 months, and WHZ for those under 61 months. Nutritional status was classified using WHO cutoffs: normal (−2SD to + 2SD), moderate undernutrition (<−2SD to −3SD), and severe undernutrition (<−3SD). We reported nutritional status previously[42].

### Ethical considerations

Ethics approval for this study was obtained from the University of Sydney Human Research Ethics Committee (HREC) (2016/456), Hanoi Medical University (HMU) (1722/QD-DHYHN) and the NCH Hanoi (812/QD-BVNTU). Written informed consent was obtained from the parents/caregivers of all study participants.

### Statistical analysis

Descriptive analyses (mean, standard deviation (SD), median and proportion) was performed. Tests of binomial proportions were conducted to determine the significance of differences between immunization coverage in the CP cohort and general child population. A *p-*value <0.05 was considered statistically significant. Logistic regression analyses were performed to determine associations between independent and outcome variables. Variables reported in other studies as factors associated with immunization status were included as independent variables[12,21,27–31]. All analyses were performed using STATA Statistics software version 16.

## Results

Data were collected from 765 children with a clinical diagnosis of CP. The median age of participants was 1.7 years (IQR = 2.7 years; range: 2.4mo - 13y 5mo) and 64.2% (n = 491) were male. The mean age at diagnosis of CP was 1 year 8 months (SD 1y 9mo).

Table 1 provides information on immunization rates among the CP cohort and the general child population in Vietnam. Among children with CP, 82.7% were fully immunized, 94% had a BCG vaccination scar and 95.9% had MR vaccination. For all these vaccines, immunization rates among the children with CP were substantially lower than for children in the general population.

**Table 1:** Immunization coverage rate among children with CP compared to the general child population in Vietnam.

Immunization variable	Children with CP immunized (%)	Vietnamese general child population immunized (%) (n = 23,371,882)[43]
Fully immunized	82.7 (n = 753)	96.4[18,20]
BCG	94.0 (n = 746)	96[18]
MR vaccine	95.9 (n = 389)^a^	98.2[35]

^a^389 children 1–14 years old during the MR campaign had available data

Various factors were found to have a significant association with full immunization status, included: older children (OR = 1.25), children born at a health care centre (OR = 3.98), home birth (OR = 0.21), living in accommodation with earth/sand floor (OR = 0.24), maternal education is Secondary/ high school completed (OR = 2.5; 2.61, respectively) (p < 0.05). Household income was not associated with immunization (table 2).

**Table 2:** Immunization status and associated socio-demographic characteristics.

Characteristics n = 765	Total n (%)	Fully immunized			Unadjusted OR for fully immunized (95% CI)	*p-*value
		No; n (%)	Yes; n (%)	*p* value		
**Age (median, IQR) (n = 753)**	1.7, 2.7	1.5, 1.5	1.8, 2.8	**<0.001**	1.25 (1.11–1.39)	**<0.001**
**Sex (n = 753)**						
Male	483 (64.1)	92 (70.8)	391 (62.8)	0.083	1	0.084
Female	207 (35.9)	38 (29.2)	232 (37.2)		1.43 (0.95-2.16)	
**Place of birth (n = 746)**						
Hospital	678 (90.9)	121 (93.8)	557 (90.3)	**<0.001**	1	
Health care centre	58 (7.8)	3 (2.3)	55 (8.9)		3.98 (1.22-12.94)	**0.022**
Home birth	10 (1.3)	5 (3.9)	5 (0.8)		0.21 (0.06-0.76)	**0.017**
**Birth order (n = 751)**						
1^st^	375 (49.9)	62 (47.7)	313 (50.4)	0.745	1	
2^nd^	260 (34.6)	45 (34.6)	215 (34.6)		0.94 (0.62-1.44)	0.798
3^rd^	101 (13.5)	19 (14.6)	82 (13.2)		0.85 (0.48-1.50)	0.589
>3^rd^	15 (2.0)	4 (3.1)	11 (1.8)		0.54 (0.16-1.76)	0.311
**Antenatal care (n = 748)**						
No	30 (4.0)	8 (6.2)	22 (3.6)	0.163	1	0.169
Yes	718 (96.0)	121 (93.8)	597 (96.5)		1.79 (0.78–4.12)	
**Type of accommodation (n = 743)**						
Finished floor	701 (94.4)	118 (92.2)	583 (94.8)	**0.019**	1	
Rough wood/bamboo house	29 (3.9)	4 (3.1)	25 (4.1)		1.26 (0.43-3.7)	0.668
Earth/sand house	13 (1.7)	6 (4.7)	7 (1.1)		0.24 (0.07-0.71)	**0.011**
**Household sanitation (n = 748)**						
Flush toilet	624 (83.4)	105 (81.4)	519 (83.8)	0.496	1	0.497
Pit toilet	124 (16.6)	24 (18.6)	100 (16.2)		0.84 (0.51-1.37)	
**Mother’s age (x ± SD) (n = 764)**	26.85 ± 5.17	26.8 ± 4.90	26.88 **± **5.24	0.954	1.00 (0.96 - 1.04)	0.875
**Mother’s education (n = 751)**						
Illiterate/ Primary school completed	37 (4.9)	12 (9.2)	25 (4.0)	0.050	1	
Secondary school completed	193 (25.7)	31 (23.9)	162 (26.1)		2.50 (1.14-5.51)	**0.022**
High school completed	393 (52.3)	61 (46.9)	332 (53.5)		2.61 (1.24-5.47)	**0.011**
Graduation/ Post graduation	128 (17.1)	26 (20.0)	102 (16.4)		1.88 (0.83-4.24)	0.127
**Father’s age (n = 755)**	30.24 ± 5.81	30.47 ± 5.69	30.20 ± 5.85	0.398	0.99 (0.96-1.02)	0.633
**Father’s education (n = 732)**						
Illiterate/ Primary completed	36 (4.9)	4 (3.1)	32 (5.3)	0.765	1	
Secondary school completed	217 (29.6)	38 (29.5)	179 (29.7)		0.58 (0.19-1.76)	0.344
Highschool completed	352 (48.1)	64 (49.6)	288 (47.8)		0.56 (0.19-1.64)	0.294
Graduation/ Post graduation	127 (17.4)	23 (17.8)	104 (17.2)		0.57 (0.18-1.75)	0.324
**Median (IQR) monthly family income (n = 727)**	87 (89)	87 (93.5)	87 (83.5)	0.315	1.00 (0.99-1.00)	0.436

In table 3, various factors related to medical history, health status/ co-morbid conditions were association with immunization status. A CP diagnosis after three years old (OR = 2.07) was associated with complete immunization. Children with bilateral CP (OR = 0.30), GMFCS level IV-V (OR = 0.25), epilepsy (OR = 0.31), intellectual/visual/speech impairments (OR: 0.54; 0.47; 0.46, respectively); and poor nutritional status (OR for WAZ, HAZ, WHZ ranged from 0.4–0.55) were less likely to be fully immunized (*p* < 0.05).

**Table 3:** Immunization status and associated factors related to medical history, health status/co-morbid conditions of children with CP.

Characteristic n = 765	Total n (%)	Fully immunized			Unadjusted OR for fully receiving immunization (CI)	*p-*value
		No n (%)	Yes n (%)	*p* value		
**Age at CP diagnosis (n = 743)**						
3 years-old and under	650 (87.5)	118 (92.9)	532 (86.4)	**0.042**	1	**0.046**
After 3 years of age	93 (12.5)	9 (7.1)	84 (13.6)		2.07 (1.01-4.23)	
**Type of CP (n = 753)**						
Unilateral	154 (20.4)	11 (8.5)	143 (23.0)	**<0.001**	1	
Bilateral	563 (74.8)	115 (88.5)	448 (71.9)		0.30 (0.16–0.57)	**<0.001**
Ataxia	1 (0.1)	1 (0.7)	0		–	–
Dyskinesia	35 (4.7)	3 (2.3)	32 (5.1)		0.82 (0.21–2.11)	0.771
**GMFCS level (n = 743)**						
I - III	400 (53.8)	35 (27.3)	365 (59.4)	**<0.001**	1	**<0.001**
IV - V	343 (46.2)	93 (72.7)	250 (40.6)		0.25 (0.16-0.39)	
**Associated impairments**						
Epilepsy (n = 747)	82 (11)	29 (22.8)	53 (8.6)	**<0.001**	0.31 (0.19-0.52)	**<0.001**
Intellectual (n = 749)	432 (57.7)	90 (69.2)	342 (55.3)	**0.003**	0.54 (0.36-0.82)	**0.004**
Visual (n = 732)	40 (5.5)	12 (9.3)	28 (4.6)	**0.035**	0.47 (0.23-0.96)	**0.038**
Hearing (n = 748)	13 (1.7)	3 (2.4)	10 (1.6)	0.555	0.67 (0.18-2.49)	0.557
Speech (n = 750)	447 (59.6)	95 (73.6)	352 (56.7)	**<0.001**	0.46 (0.31-0.71)	**<0.001**
**Weight-for-Age Z Score (n = 727)**						
Normal	516 (71)	70 (54.3)	446 (74.6)	**<0.001**	1	**0.001**
Underweight	211 (29)	59 (45.7)	152 (25.4)		0.40 (0.27-0.59)	
**Height-for-Age Z Score (n = 706)**						
Normal	435 (61.6)	58 (49.6)	377 (64)	**0.003**	1	**0.004**
Stunted	271 (38.4)	59 (50.4)	212 (36)		0.55 (0.37-0.82)	
**Weight-for-Height Z Score (n = 572)**						
Normal	391 (68.4)	60 (55.6)	331 (71.3)	**0.001**	1	**0.002**
Wasted	181 (31.6)	48 (44.4)	133 (28.7)		0.5 (0.32-0.77)	

In the multivariate logistic model, rough wood/bamboo accommodation (OR = 15.01) and higher levels of maternal education (OR=~6) were associated with full immunization whereas GMFCS level IV – V (OR = 0.37) and having epilepsy (OR = 0.27) were associated with incomplete immunization among children with CP (Table 4).

**Table 4:** Immunization status and associated factors (adjusted OR multivariate logistic model).

Characteristic (n = 467)	Adjusted OR for receipt of full immunization	95% CI
**Age**	0.94	0.72 - 1.24
**Female**	1.52	0.85 - 2.71
**Place of birth**		
Hospital	1	
Health care center	7.59	0.87 - 65.87
Home birth	0.31	0.04 - 2.19
**Birth order**		
1st	1	
2nd	1.04	0.53 - 2.02
3rd	1.48	0.53 - 4.16
>3rd	0.97	0.14 - 6.75
**Antenatal care (Yes vs. No)**	1.34	0.23 - 7.78
**Type of accommodation**		
Finished floor	1	
Rough wood/bamboo	**15.01**	**1.19 - 189.75**
Earth, sand	0.44	0.09 - 2.18
**Household sanitation (**Pit toilet vs. Flush toilet)	1.19	0.54 - 2.61
**Mother’s age**	1.03	0.94 - 1.13
**Mother’s education**		
Illiterate/ Primary school completed	1	
Secondary school completed	**6.54**	**1.54 - 27.65**
High school completed	**5.95**	**1.37 - 25.77**
University Graduate/Post-graduate	**5.71**	**1.08 - 30.29**
**Father’s age**	0.98	0.91 - 1.06
**Father’s education**		
Illiterate/ Primary completed		
Secondary school completed	0.43	0.08 - 2.26
Highschool completed	0.33	0.06 - 1.74
Graduation/ Post-graduation	0.49	0.07 - 3.25
**Monthly family income (USD)**	1.00	1.00 - 1.01
**Age at CP diagnosis after 3 years old**	0.42	0.12 - 1.45
**Type of CP**		
Unilateral	1	
Bilateral	0.63	0.27 - 1.45
Ataxia	–	–
Dyskinesia	0.48	0.07 - 3.09
**GMFCS level IV – V**	**0.37**	**0.19 - 0.71**
**Associated impairments (Yes vs. No)**		
Epilepsy (n = 747)	**0.27**	**0.12 - 0.58**
Intellectual (n = 749)	0.91	0.50 - 1.68
Visual (n = 732)	0.83	0.30 - 2.33
Hearing (n = 748)	3.04	0.26 - 35.09
Speech (n = 750)	0.94	0.50 - 1.75
**Weight-for-Age Z Score** (Underweight vs. normal)	0.65	0.36 - 1.20
**Height-for-Age Z Score** (Stunted vs. normal)	0.86	0.49 - 1.51
**Weight-for-Height Z Score** (Wasted vs. normal)	0.77	0.44 - 1.36

## Discussion

In this study, we investigated factors associated with immunization status among children with CP in Vietnam. Our findings confirm that rates of immunization among children with CP, even in a hospital-based cohort, are lower than in their peers in the general child population. This is consistent with several other studies that suggest that children with CP and other chronic neurological conditions are less likely to receive timely vaccination compared to the general child population[12,21,24,31,32,44]. The proportion of children with CP in our study who were fully immunized according to the EPI for Vietnam was comparable to findings from a study reporting immunization rates among children with CP in Australia (80.8%)[12]. However, the rate was significantly lower than in children with disabilities or chronic neurological diseases in Turkey (95.6%)[25] and Ecuador (96.9%)[13]. The high immunization rate observed in Ecuador is underscored by the outstanding disability care in the country, including free healthcare in public hospitals provided under a national insurance program[45] and home-based health and rehabilitation services[13]. There are opportunities to enhance immunization coverage among children with disabilities in Vietnam by implementing similar policies and interventions.

The proportion of children with a BCG vaccination scar in our hospital-based study is comparable to that in some population-based CP cohorts. For example, in Bangladesh the rate was 92.3%[21] and in Turkey 90.8%[23]. The BCG rate was lower in Vietnam, however than in a population based CP cohort in China (98.4%)[22]. In contrast, a study in the remote island of Sumba in Indonesia reported a BCG vaccination rate of 73.1% among children with CP. [24] Children with CP are at greater risk of infections, thus are more likely to be unwell at the time of vaccinations in the early years of life. This can be an additional barrier to immunization among these children. [31].

In our study, the rate of MR vaccination was significantly lower than in the general child population but within the 43.2% to 96.1% range observed among children with disabilities (that include CP and chronic neurological diseases) in Vietnam[12,21–23,25]. This suggests that there are additional barriers to routine immunization for children with CP during the post-neonatal period.

### Factors associated with immunization status

In our study, the proportion of children who were fully immunized according to the EPI was significantly higher among children with CP who were older (>3 years) at diagnosis (*p* = 0.046). A similar trend was reported by the ACPR[12] which showed that younger children were more likely to be overdue for vaccination. This highlights the importance of focused interventions to enhance immunization coverage early in life and minimise missed opportunities. A proportion of children diagnosed with CP later in life have a mild type of CP (i.e., GMFCS level I-II, no/mild impairment), thus are more easily able to access healthcare, including the national EPI, alongside their peers without disability. Interestingly, data from the BCPR shows that children with CP who were diagnosed before three years of age were 2.7 times more likely to have been vaccinated against rubella than children diagnosed after three years of age[21]. This reflects that an early diagnosis could facilitate early entry into the healthcare system and more timely access to vaccines.

Some variables in our multivariate model had wide confidence intervals. For example, we found that the level of maternal education was independently associated with full immunization status, similar to studies in general child populations in Nepal, Bangladesh, India and Indonesia[27–30]. However, the wide confidence intervals for this variable suggest underpowering, likely due to variability in the data, which may reduce the precision of estimates. This study did not account for potential clustering effects, such as geographic or socio-economic factors, which may influence immunization status. We acknowledge that use of more refined models (e.g., hierarchical or multilevel modeling) could improve precision. Mothers with higher level of education are likely to be more health literate and aware of the benefits and importance of vaccination of their children. Health education efforts should ensure that mothers with lower levels of education are educated about child vaccination and helped to access services.

Several previous studies, both in children with CP and in the general population, report the link between socioeconomic status and childhood immunization[21,27,30]. In our study, no association was observed between the monthly household income and immunization status of children with CP. This is possibly because EPI vaccines are available free of cost in Vietnam. Furthermore, free disclosure of information about income is uncommon in Vietnamese culture and this could have introduced bias in the data we obtained. However, accommodation type (wood/bamboo versus earth/sand house), a proxy for income, was associated with complete vaccination of children with CP. Similarly, families living in earth or sand houses are often socioeconomically disadvantaged and live in rural or remote areas which limit access to health services such as immunization. The wide confidence intervals for this variable also suggest underpowering, likely caused by data variability, which may reduce the precision of the estimates.

Regarding nutritional status, children with CP who were underweight, stunted or wasted were less likely to be fully vaccinated (*p* < 0.01). Poor nutritional status of children may reflect socioeconomic disadvantage and is both a potential cause and consequence of CP[34]. Socioeconomic disadvantage per se is associated with increased risk of CP and contributes to more severe functional limitations owing to greater exposure to risk factors and limited access to healthcare. Furthermore, these families may lack access to health education and resources that highlight the key role of good nutrition and other health intervention such as immunization for child wellbeing[46,47].

Place of birth was an important factor associated with immunization in this study. In all hospitals and health care centers in Vietnam, Hepatitis B and BCG vaccines are administered immediately after birth according to the EPI[48]. Therefore, the significantly lower rate of complete immunization among those who were born at home is unsurprising. Similar findings were reported in a study on immunization among the general child population in Nepal[28].

Children with bilateral CP and motor function at GMFCS levels IV-V were less likely to be fully immunized than those with unilateral CP and function at GMFCS levels I-III respectively. This indicates that more severe impairment may limit access to immunization services. Furthermore, associated impairments including epilepsy and intellectual, visual and speech impairment, were associated with incomplete immunization. A previous study found that the MMR vaccination rate was lower among children with epilepsy than those without[32]. These findings highlight the importance of focused interventions for vaccination of children with severe motor and other impairments associated with CP. Strategies might include outreach programs providing vaccines via home visits, frequent clinician follow up of children with disability, and sending vaccination reminders to caregivers.

There was no association observed between birth order and immunization status in our study. Some studies report that parents who experienced any complications during the birth of their first child are less likely to vaccinate subsequent children[49,50]. Vaccination consultations should emphasize the benefits of disease prevention while describing potential side effects. Antenatal care was not found to significantly impact immunization in our study, although some studies conducted in general populations have reported that inadequate antenatal care adversely influenced completeness of child immunization[27,29]. Ensuring equity of access to immunization is crucial for children with CP to prevent further disability and optimise quality of life. We recommend that prenatal clinics provide resources for parents such as brochures and that clinicians advice all pregnant mothers about the importance of timely and complete vaccination of their children. Furthermore, many LMICs lack data on immunization uptake in children with disabilities. This novel Vietnamese data could inform future research and strategies to improve vaccination uptake in other LMICs.

### Study limitations

Despite our considerable efforts, we acknowledge some important limitations in our study. Firstly, relying on historical data to determine full immunization status introduces uncertainty into our results, despite the relatively short historical timeframe (a young cohort with a median age of 1.7 years) and completion of the national MR campaign only a year prior. Also, data collection on some factors relied on parent/caregivers report which may be subject to recall bias. Secondly, we acknowledged the potential selection bias in our hospital-based cohort. Children who seek care at a tertiary hospital may have more severe disease, greater access to healthcare or higher socioeconomic backgrounds, while children with milder CP or those facing barriers to healthcare access may be underrepresented.

Thirdly, qualitative exploration of certain factors that potentially influence immunization of children with CP was beyond the scope of this study. This includes social, cultural beliefs, health seeking behaviour, vaccine hesitancy, systemic barriers and stigma associated with disability[51]. A potential limitation of our study is the lack of information on cultural beliefs and practices among the minority groups included in our sample. Additionally, the stigma associated with disabilities may influence some caregivers’ willingness to seek and engage with immunization programs. However, we did not collect data from parents regarding potential stigma related to seeking healthcare. Thus, the findings must be interpreted with caution and may not be representative of children with CP in other settings in Vietnam. Other systemic barriers include a lack of disability-inclusive services, financial constraints, and high caregiving demands, which also make it difficult for parents to prioritize vaccinations.

## Conclusion

To our knowledge, this is the first study to provide robust evidence on the immunization status and associated factors among children with CP in Vietnam. The rate of full immunization among children with CP in Vietnam was significantly lower than in children in the general population and in children with CP in other countries. The major factors influencing the lower rates of immunization in CP included: younger age, living in an earth/sand house, being born at home, having a mother with a low level of education, age at CP diagnosis under three years old, bilateral, having associated impairments (i.e., epilepsy, intellectual, visual, speech), a GMFCS level of IV-V and undernutrition. To increase vaccination coverage, interventions and strategies are required to ensure that all children with CP have equitable access to early diagnosis, immunization, health education programs, outreach programs, and frequent follow-up. Early diagnosis and focused intervention in early life could further improve vaccination coverage in children with CP. The interventions could emphasize mobile immunization units to enable immunization for children with severe CP in their home and provide transportation support for families of children with CP to overcome mobility barriers. Such interventions would contribute to a positive and long-term improvement in the health and well-being of children with CP in Vietnam and neighbouring countries with similar context.

## Supporting information

S1 File STROBE checklist. (DOCX)

S2 File Inclusivity in global research. (DOCX)

S3. File Data. (XLSX)

## References

[1] World Health Organization. *Essential Programme on Immunization*. Available at https://www.who.int/teams/immunization-vaccines-and-biologicals/essential-programme-on-immunization, accessed on 14/12/2022

[2] UNICEF. Convention on the Rights of the Child. 1989.

[3] Training Guide. The convention on the rights of persons with disabilities. New York and Geneva: United Nations of Human Rights, 2014.

[4] March B, Eastwood K, Wright IM, Tilbrook L, Durrheim DN. Epidemiology of enteroviral meningoencephalitis in neonates and young infants. J Paediatr Child Health. 2014;50(3):216–20. doi: 10.1111/jpc.12468 24372592

[5] Tebruegge M, Curtis N. Enterovirus infections in neonates. Semin Fetal Neonatal Med. 2009;14(4):222–7. doi: 10.1016/j.siny.2009.02.002 19303380

[6] Ostrander B, Bale JF. Congenital and perinatal infections. Handb Clin Neurol. 2019;162:133–53. doi: 10.1016/B978-0-444-64029-1.00006-0 31324308

[7] Unicef. *UNICEF Fact Sheet: Children with Disabilities* (available at https://www.unicef.org/media/128976/file/UNICEF%20Fact%20Sheet%20:%20Children%20with%20Disabilities.pdf, accessed on 22 Jan 2024). 2022.

[8] Havers F, Fry AM, Chen J, Christensen D, Moore C, Peacock G, et al. Hospitalizations attributable to respiratory infections among children with neurologic disorders. J Pediatr. 2016;170:135-41.e1-5. doi: 10.1016/j.jpeds.2015.11.030 26687576

[9] Jahan I, Karim T, Das MC, Muhit M, McIntyre S, Smithers-Sheedy H, et al. Mortality in children with cerebral palsy in rural Bangladesh: a population-based surveillance study. Dev Med Child Neurol. 2019. 61(11): p. 1336–43. 31081134 10.1111/dmcn.14256

[10] Maulik PK, Darmstadt GL. Childhood disability in low- and middle-income countries: overview of screening, prevention, services, legislation, and epidemiology. Pediatrics. 2007;120(Suppl 1):S1-55. doi: 10.1542/peds.2007-0043B 17603094

[11] O’Neill J, Newall F, Antolovich G, Lima S, Danchin M. Vaccination in people with disability: a review. Hum Vaccin Immunother. 2020;16(1):7–15. doi: 10.1080/21645515.2019.1640556 31287773 PMC7012164

[12] Greenwood VJ, Crawford NW, Walstab JE, Reddihough DS. Immunisation coverage in children with cerebral palsy compared with the general population. J Paediatr Child Health. 2013;49(2):E137-41. doi: 10.1111/jpc.12097 23360148

[13] Groce NE, Ayora P, Kaplan LC. Immunization rates among disabled children in Ecuador: unanticipated findings. J Pediatr. 2007;151(2):218–20. doi: 10.1016/j.jpeds.2007.04.061 17643783

[14] Bazzano A, Zeldin A, Schuster E, Barrett C, Lehrer D. Vaccine-related beliefs and practices of parents of children with autism spectrum disorders. Am J Intellect Dev Disabil. 2012;117(3):233–42. doi: 10.1352/1944-7558-117.3.233 22716265

[15] Kapuria B, Hamadeh RS, Mazloum F, Chaalan K, Aung K, Higgins E, et al. Immunization as an entry point for primary health care and beyond healthcare interventions-process and insights from an integrated approach in Lebanon. Front Health Serv. 2023;3:1251775. doi: 10.3389/frhs.2023.1251775 37965097 PMC10641862

[16] Vietnam National Institute of Hygiene and Epidemiology. *Results of the Expanded Program in Immunization*. Available at http://www.tiemchungmorong.vn/vi/content/thanh-qua.html. Accessed on 23/9/2022 [in Vietnamese].

[17] Ministry of Health Vietnam. National Expanded Program on Immunization: Comprehensive Multi-Year Plan for Extended Program on Immunization 2016-2020. 2015: Ministry of Health Vietnam.

[18] Nguyen CTT, Grappasonni I, Scuri S, Nguyen BT, Nguyen TTT, Petrelli F. Immunization in Vietnam. Ann Ig, 2019;31(3):291–305. 31069373 10.7416/ai.2019.2291

[19] Prevention; V.C.f.D.C.a. *Summary of the Measles-Rubella Vaccination Campaign for Children Aged 1 to 14 Years* (available at https://vncdc.gov.vn/gap-go-bao-chi-tong-ket-chien-dich-tiem-vac-xin-soi-rubella-cho-tre-tu-1-den-14-tuoi-nd14082.html, accessed on 16 March 2024) [in Vietnamese]. 2015.

[20] Nguyen TD, Dang AD, Van Damme P, Nguyen CV, Duong HT, Goossens H, et al. Coverage of the expanded program on immunization in Vietnam: Results from 2 cluster surveys and routine reports. Hum Vaccin Immunother. 2015;11(6):1526–33. doi: 10.1080/21645515.2015.1032487 25970593 PMC4514219

[21] May P, Smithers-Sheedy H, Muhit M, Cumming R, Jones C, Booy R, et al. Immunisation status of children with cerebral palsy in Rural Bangladesh: results from the Bangladesh Cerebral Palsy Register (BCPR). Infect Disord Drug Targets. 2020;20(3):318–22. doi: 10.2174/1871526518666181024101002 30360749

[22] Yang L, Peng J, Deng J, He F, Chen C, Yin F, et al. Vaccination status of children with epilepsy or cerebral palsy in hunan rural area and a relative KAP survey of vaccinators. Front Pediatr. 2019;7:84. doi: 10.3389/fped.2019.00084 30984716 PMC6448507

[23] Bozkaya-Yilmaz S, Karadag-Oncel E, Olgac-Dundar N, Gencpinar P, Sarioglu B, Arican P, et al. Evaluation of immunization status in patients with cerebral palsy: a multicenter CP-VACC study. Eur J Pediatr. 2022;181(1):383–91. doi: 10.1007/s00431-021-04219-4 34355277

[24] Jahan I, Al Imam MH, Karim T, Muhit M, Hardianto D, Das MC, et al. Epidemiology of cerebral palsy in Sumba Island, Indonesia. Dev Med Child Neurol, 2020;62(12):1414–22. 32686098 10.1111/dmcn.14616

[25] Dinleyici M, Carman KB, Kilic O, Laciner Gurlevik S, Yarar C, Dinleyici EC. The immunization status of children with chronic neurological disease and serological assessment of vaccine-preventable diseases. Hum Vaccin Immunother. 2018;14(8):1970–6. doi: 10.1080/21645515.2018.1460986 29624477 PMC6149706

[26] Okoro JC, Ojinnaka NC, Ikefuna AN, Onyenwe NE. Sociodemographic influences on immunization of children with chronic neurological disorders in Enugu, Nigeria. Trials Vaccinol. 2015;4:9–13. doi: 10.1016/j.trivac.2014.11.002

[27] Wahl B, Gupta M, Erchick DJ, Patenaude BN, Holroyd TA, Sauer M, et al. Change in full immunization inequalities in Indian children 12-23 months: an analysis of household survey data. BMC Public Health. 2021;21(1):841. doi: 10.1186/s12889-021-10849-y 33933038 PMC8088616

[28] Patel PN, Hada M, Carlson BF, Boulton ML. Immunization status of children in Nepal and associated factors, 2016. Vaccine. 2021;39(40):5831–8. doi: 10.1016/j.vaccine.2021.08.059 34456076

[29] Siramaneerat I, Agushybana F. Inequalities in immunization coverage in Indonesia: a multilevel analysis. Rural Remote Health. 2021;21(3):6348. 34432982 10.22605/RRH6348

[30] Banerjee S, SubirBiswas, Roy S, Pal M, Hossain MG, Bharati P. Nutritional and immunization status of under-five children of India and Bangladesh. BMC Nutr. 2021;7(1):77. doi: 10.1186/s40795-021-00484-6 34852848 PMC8638544

[31] Pandolfi E, Carloni E, Marino MG, Ciofi degli Atti ML, Gesualdo F, Romano M, et al. Immunization coverage and timeliness of vaccination in Italian children with chronic diseases. Vaccine. 2012;30(34):5172–8. doi: 10.1016/j.vaccine.2011.02.099 21414380

[32] Gitiaux C, Poeyo S, Demazes S, Pedespan L, Pillet P, Husson M, et al. Vaccinal status in children with seizures: a retrospective study conducted in the Bordeaux child hospital. Arch Pediatr. 2006;13(8):1102–6. doi: 10.1016/j.arcped.2006.04.008 16697162

[33] Khandaker G, Van Bang N, Dũng TQ, Giang NTH, Chau CM, Van Anh NT, et al. Protocol for hospital based-surveillance of cerebral palsy (CP) in Hanoi using the Paediatric Active Enhanced Disease Surveillance mechanism (PAEDS-Vietnam): a study towards developing hospital-based disease surveillance in Vietnam. BMJ Open. 2017;7(11):e017742. doi: 10.1136/bmjopen-2017-017742 29127227 PMC5695473

[34] Karim T, Dossetor R, Huong Giang NT, Dung TQ, Son TV, Hoa NX, et al. Data on cerebral palsy in Vietnam will inform clinical practice and policy in low and middle-income countries. Disabil Rehabil, 2021;1–8. 33397164 10.1080/09638288.2020.1854872

[35] General department of Preventive medicine Vietnam. Summary of measles-rubella vaccination campaign for children aged 1 to 14 years [in Vietnamese]. 2015.

[36] Rosenbaum PL, Palisano RJ, Bartlett DJ, Galuppi BE, Russell DJ. Development of the gross motor function classification system for cerebral palsy. Dev Med Child Neurol. 2008;50(4):249–53. doi: 10.1111/j.1469-8749.2008.02045.x 18318732

[37] Schalock RL, Borthwick-Duffy SA, Bradley VJ, Buntinx WH, Coulter DL, Craig EM, et al. Intellectual disability: Definition, classification, and systems of supports. 2010: ERIC.

[38] World Health Organization. *Blindness and vision impairment* [availble at https://www.who.int/news-room/fact-sheets/detail/blindness-and-visual-impairment, accessed on 10 Jul 2017).

[39] World Health Organization. *Hearing screening: considerations for implementation* [available at https://www.who.int/publications/i/item/9789240032767, accessed on 10 Jul 2022).

[40] Khuc THH, Karim T, Nguyen VAT, Giang NTH, Dũng TQ, Dossetor R, et al. Associated impairments among children with cerebral palsy: findings from a cross-sectional hospital-based study in Vietnam. BMJ Open. 2024;14(10):e075820. doi: 10.1136/bmjopen-2023-075820 39461866 PMC11535665

[41] World Health Organization, WHO Global Database on Child Growth and Malnutrition; World Health Organization: Geneva, Switzerland. Available online: http://apps.who.int/iris/bitstream/10665/63750/1/WHO_NUT_97.4.pdf (accessed on 8/8/2022). 1997.

[42] Karim T, Jahan I, Dossetor R, Giang NTH, Van Anh NT, Dung TQ, et al. Nutritional status of children with cerebral palsy-findings from prospective hospital-based surveillance in Vietnam indicate a need for action. Nutrients. 2019;11(9):2132. doi: 10.3390/nu11092132 31500109 PMC6769778

[43] General Statistics Office. *Completed results of the 2019 Viet Nam population and housing census* (avaiable at https://www.gso.gov.vn/wp-content/uploads/2019/12/Ket-qua-toan-bo-Tong-dieu-tra-dan-so-va-nha-o-2019.pdf, accessed on 07 Jul 2023). 2020.

[44] Jahan I, Al Imam MH, Muhit M, Chhetri AB, Badawi N, Khandaker G. Epidemiology of cerebral palsy among children in the remote Gorkha district of Nepal: findings from the Nepal cerebral palsy register. Disabil Rehabil, 2022:1–10. 10.1080/09638288.2022.2118871 36102553

[45] World Health Organization. Country Cooperation Strategy at a Glance: Ecuador, in 2 p. Report No.: 1. 2013.

[46] Ibrahim MMK, Loubou ML, Luc-Aime KS, Aminou M, Marcellin NG. Influence of socioeconomic factors on the nutritional status of pupils of 5 to 11 in rural areas in cameroon: case of the Nyambaka municipality in the Adamawa region. Indian J Public Health. 2022;66(2):193–5. doi: 10.4103/ijph.ijph_1516_21 35859505

[47] Aheto JMK, Pannell O, Dotse-Gborgbortsi W, Trimner MK, Tatem AJ, Rhoda DA, et al. Multilevel analysis of predictors of multiple indicators of childhood vaccination in Nigeria. PLoS One. 2022;17(5):e0269066. doi: 10.1371/journal.pone.0269066 35613138 PMC9132327

[48] Ministry of Health Vietnam. Circular 38/2017/TT-BYT on the list of communicate diseases, scope and subjects must use vaccines and medical biological products [in Vietnamese]. 2017.

[49] Galadima AN, Zulkefli NAM, Said SM, Ahmad N. Factors influencing childhood immunisation uptake in Africa: a systematic review. BMC Public Health. 2021;21(1):1475. doi: 10.1186/s12889-021-11466-5 34320942 PMC8320032

[50] Bbaale E. Factors influencing childhood immunization in Uganda. J Health Popul Nutr. 2013;31(1):118–29. doi: 10.3329/jhpn.v31i1.14756 23617212 PMC3702366

[51] Norman G, Kletter M, Dumville J. Interventions to increase vaccination in vulnerable groups: rapid overview of reviews. BMC Public Health. 2024;24(1):1479. doi: 10.1186/s12889-024-18713-5 38831275 PMC11145854

